# Comparison of two approaches to measuring clean faces as part of the facial cleanliness component of the SAFE trachoma elimination strategy

**DOI:** 10.1371/journal.pntd.0012090

**Published:** 2024-04-10

**Authors:** Sheila K. West, Beatriz Munoz, Harran Mkocha, Matthew C. Lynch, Catherine Gracewello, Mabula Kasubi, Meraf A. Wolle

**Affiliations:** 1 Dana Center for Preventive Ophthalmology, Wilmer Eye Institute, Johns Hopkins Medicine, Baltimore, Maryland, United States of America; 2 Kongwa Trachoma Project, Kongwa, Tanzania; 3 Muhimbili Medical Center, Dar Es Salaam Tanzania; Carter Center, UNITED STATES

## Abstract

**Background:**

The Alliance for the Global Elimination of Trachoma (GET) endorses the full SAFE strategy to eliminate trachoma; Surgery (for trichiasis), Antibiotics (to reduce the community pool of infection, Facial cleanliness, and Environmental improvement (to decrease transmission). There is no accepted measure of facial cleanliness. This study compared two possible metrics for facial cleanliness.

**Method/Findings:**

Metric one: Clean face was defined as observed absence of ocular and nasal discharge on the face. Metric two: observing a grade of dirtiness (scale 10 = lightest to 0 = darkest) on a standard facial wipe. The reliability of grading a child’s face or grading a facial wipe was determined in children in Kongwa Tanzania. We also observed both measurements in a cohort of 202 children ages 1 to <7years prior to face cleaning, immediately afterwards, and 4 hours afterwards. Fifty of the children did not have face cleaning and were controls. Intra-and interobserver reliability was similar for both measures, the latter = 0.53 for observing a clean face and 0.52 for grading a facial wipe. There was no correlation between the two. Both measures detected facial cleaning, compared to control children who were not cleaned, immediately after cleaning; control children with 53% clean faces and wipe score of 6.7 compared to cleaned children with 88% clean faces and wipe score of 8 (p = .0001, p = < .0001, respectively). Both measures also detected face washing 4 hours previously compared to controls.

**Conclusions:**

The two metrics were equally reliable, and both measured the behavior of face washing. They measure different aspects of a clean face; one measures the amount of dirt on wiped area and the other measures ocular and nasal discharge. Both measurements appear to capture the behavior of facial cleaning, and the choice of metric would appear to rest on the measurement that captures the stated objective of the behavior, consideration of costs, training, logistics, and implementation.

## Author summary

Trachoma, a chronic ocular infection caused by *Chlamydia trachomatis*, is the leading infectious cause of blindness globally. The full SAFE strategy is implemented by affected countries to eliminate trachoma; Surgery (for trichiasis), Antibiotics (to reduce the community pool of infection, Facial cleanliness, and Environmental improvement (to decrease transmission). However, there is no accepted measure of facial cleanliness that programs can use to chart progress in improving hygiene. This study compared two possible metrics for facial cleanliness, observing a clean face and observing dirt on a facial wipe.

A clean face was defined as observed absence of ocular and nasal discharge on the face or observing a low grade of dirtiness (scale 10 = white to 0 = black) on a standard facial wipe. The reliability of grading a child’s face or grading a facial wipe was determined in children in Kongwa Tanzania. Both measurements were equally reliable. We tested both measures in a cohort of 202 children ages 1 to <7years prior to face cleaning, immediately afterwards, and 4 hours afterwards. There was no correlation between the two. Both measures detected facial cleaning immediately after cleaning, compared to control children who were not cleaned; control (uncleaned) had 53% clean faces and wipe score of 6.7 compared to cleaned children with 88% clean faces and wipe score of 8 (p = .0001, p = < .0001, respectively). Both measures also detected face washing 4 hours previously compared to controls.

The two metrics were equally reliable, and both measured the behavior of face washing. They measure different aspects of a clean face; one measures dirtiness and the other measures potentially infected ocular and nasal discharge. Both measurements appear to capture the behavior of facial cleaning, and the choice of metric would appear to rest on the measurement that captures the stated objective of the behavior, consideration of costs, training, logistics, and implementation.

## Introduction

Trachoma is the leading infectious cause of blindness world-wide, and a target for elimination by the World Health Organization [1]. The Alliance for the Global Elimination of Trachoma (GET) endorses the full SAFE strategy to eliminate trachoma; Surgery (for trichiasis), Antibiotics (to reduce the community pool of infection, Facial cleanliness (to decrease transmission), and Environmental improvement (to decrease transmission) [1]. Surveys to monitor the implementation of SAFE currently measure clinical signs of trachoma (trichiasis and Trachomatous inflammation—Follicular) and some measures of the household or community environment, such as larine coverage. There is no widely accepted measure of facial cleanliness.

Trachoma is spread through contact with infected ocular and nasal secretions as there is no intermediate host for the agent, *C*. *trachomatis* [1]. Therefore, researchers developed a standard observation of clean faces that reflect the presence or absence of these secretions on the face [2]. In the past, the presence of flies on the face was also included but was dropped due to transient nature of flies on the face and the impact of measurement conditions on ascertaining flies. Graders of clean faces have been shown to be reliable [2] and a clinical trial found evidence that improving clean faces resulted in decreasing prevalence of severe trachoma [3].

However, the observational metric has been criticized as not quantitative and thus insensitive to incremental changes in behavior [4]. There is also the concern that graders of clean faces may be influenced by the appearance of others in the household or the general conditions in the household or community. To this end, a new approach was developed using sterile individual saline facial wipes to measure the degree of dirt on the face against an eleven-step colorimetric chart [4].

The purpose of this study was to compare the measurement of both observed clean faces and observed degree of dirt as represented by the color of the facial wipe in a sample of 50 children ages 1-<7 from each of four communities in Kongwa Tanzania. Observations would be made prior to mothers washing the faces, immediately afterwards, and 4 hours afterwards. We hypothesized that both measures would be sensitive to face washing, but that both measures would return to pre-washed levels after 4 hours.

## Methods

### Ethics statement

All study protocols for the reliability testing and the main study were reviewed and approved by the Institutional Review Board of Johns Hopkins Medicine, and by the National Institute for Medical Research in Dar Es Salaam. Guardians provided written, informed consent for children to participate.

### Observations of clean faces

The definition of a “clean face” was the absence of ocular and nasal discharge as observed on the child’s face. We followed a previous protocol in operationalizing the definition [2]. In summary, the child’s face was defined as the area on direct frontal examination from hairline to chin, and ear to ear, excluding hair, under the chin, and neck. The face was to be examined in indirect sunlight, and signs observed in the absence of crying, which can distort the observations. Ocular discharge was defined as the presence on the lid margin or lid of clear or cloudy fluid or dry matter. Nasal discharge was defined as the presence of wet or dry discharge that is visible outside the nares. The examiner must not stare up the nostril but observe discharge in frontal view.

2 hours of training was provided on assessment of clean faces, which included observing a power point presentation and examples of clean and unclean faces, plus open discussion of clean faces on five children.

### Facial wipes and observation of degree of color

A trained team member wiped the skin around the right eye of each child at baseline, left eye at time 1 (immediately after washing) and right eye again at time 2 (4 hours after washing), using a new individually wrapped, saline sterile gauze pad for each child at each time point. The team member used a standard procedure to trace the gauze pad along the skin from the tragus of the right ear along the zygomatic bone, over the right eyelid [below the superior orbital margin], down along the line of the tear duct, infra-orbital margin, and back to the tragus of the right ear. The trained wipe grader used a standardized, 11-point brown color scale to score the color of the darkest point within the darkest half-square inch (i.e., thumb-nail sized area) of each gauze pad, per her own designation. The scale ranges in one unit from 0 (darkest brown/black) to 10 (white) (see figure in reference 4 which shows the scale. For copyright reasons, we cannot reproduce the figure).

The team member who wiped the eye was trained by MW using a guidance video supplied by Emory University. The team member who was trained as a face wipe grader was trained and standardized against MW using the colorimetric scale which was sealed in plastic to avoid getting dirty throughout the survey.

### Preliminary study

**Fig 1:**
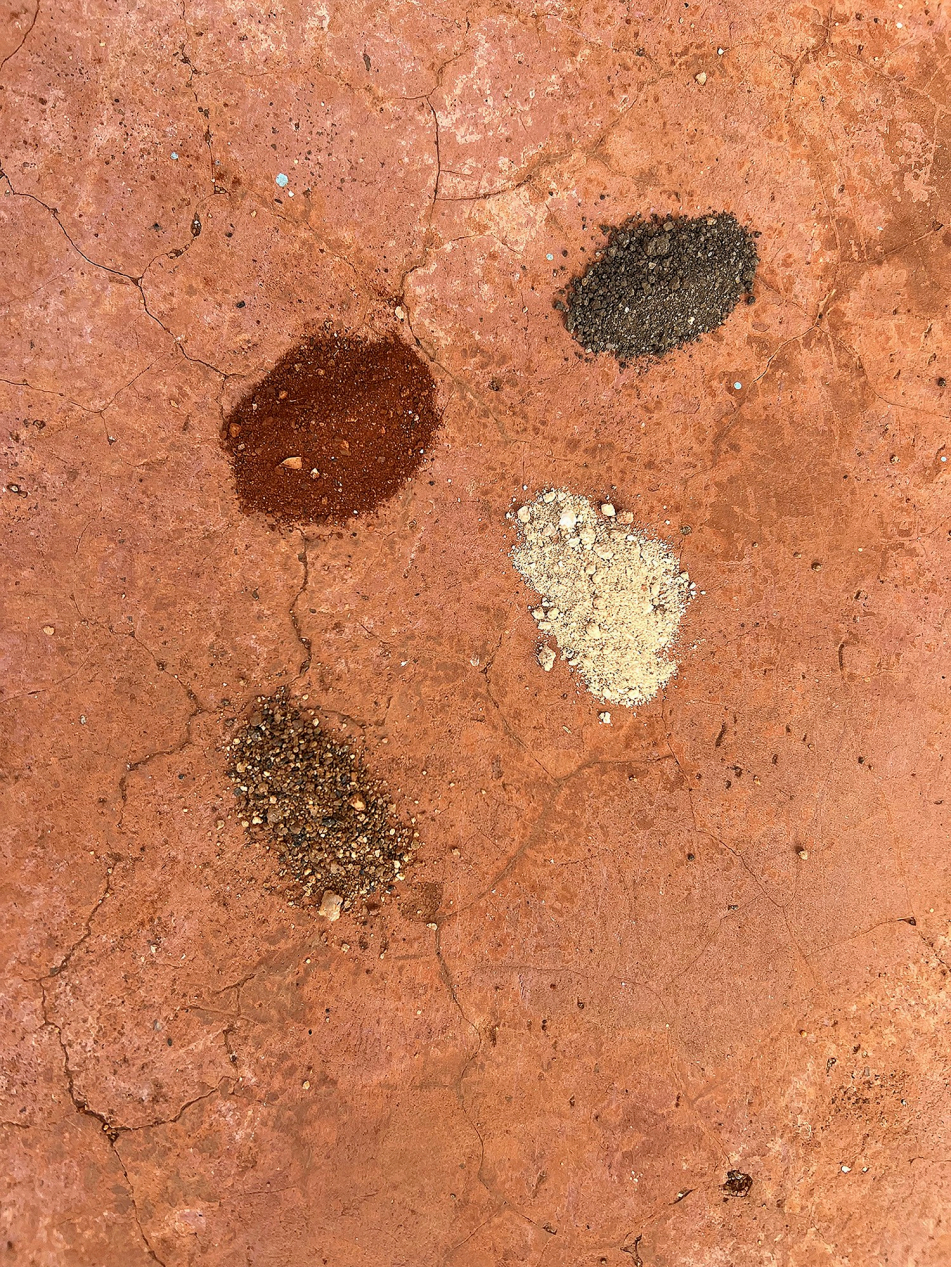
Different colors of dirt in Kongwa Tanzania.

A preliminary study was conducted measuring the effect of different colors of dirt, all common in Kongwa district and available at the study office, on the grading of wipes (see Fig 1). A standard 1/2 tsp of 3 different colors of available dirt, ground to the same consistency, was applied to the same area of the hand, which was washed and dried before and after each application. The wipe was applied as directed in protocol to the area, and then graded by SW without knowledge of the color of the dirt that was applied.

### Reliability testing

A community which was not part of the four selected below was chosen for testing the reliability of seven graders to assess facial cleanliness and to grade the color on the face wipes. A few days before, a team member visited the community and, together with the local village health worker, arranged for mothers of children to meet at a central health post on a given day to undertake inter and intraobserver variation studies. Mothers were instructed not to wash their children’s faces before arrival.

On the study day, raters were positioned at least six feet apart, but in spaces receiving a similar amount of light. The children were circulated amongst the raters who assessed their facial cleanliness, per the definition above. Raters entered their data onto paper data collection forms which were collected as soon as the child was seen. As soon as all children cycled through all raters, they were recycled back through the raters a second time. The time between the two grades for the same child was approximately ½ hour. We observed that some mothers wiped their children’s faces between times one and two, despite instructions not to do so. The children of these mothers were not excluded from the analyses.

After the child was seen twice, they were directed to a face wipe station, where MW took the face wipes on 91 children as described above. The wipe was then placed and sealed in a clear bag marked with an ID number that corresponded to the facial cleanliness assessment of each child.

At the end of the day, the same seven face graders then graded each wipe, which was laid out on a table in the office with uniform illumination. Each grader used a standardized, 11-point brown color scale described above to score the color of the darkest point within the darkest half-square inch (i.e., thumb-nail sized area) of each gauze pad. All grades were recorded on a paper grading sheet and turned in after all wipes were graded. When everyone had finished, all graders were given another grading sheet and asked to regrade the wipes as above. It was noted that one grader appeared to be copying the grades of another grader, which was confirmed, and the grader was dismissed from the study and her grades were not used in the analyses of reliability.

### Study population

**Fig 2:**
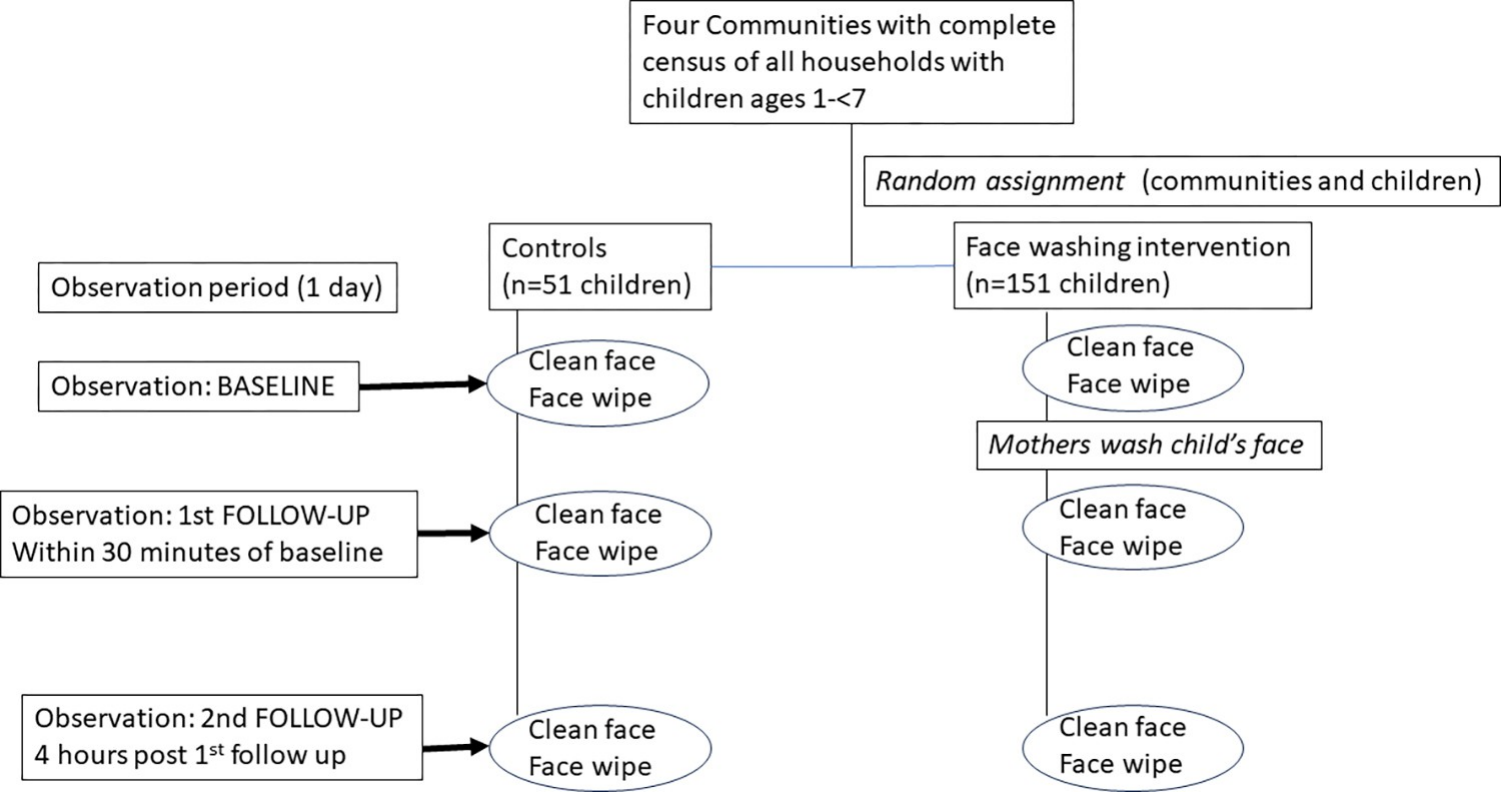
Diagram of study design and observation points for comparison study.

For the comparison study, we selected four communities and randomly selected 50–51 children from each community as described below. The communities were randomly assigned to either control (no face washing at any observation point) or one of the three intervention communities. The design and observation points are diagrammed in Fig 2.

For study budget and logistic reasons, four communities in Kongwa district were chosen based on being within one hour drive from study headquarters (as noted below, the team had to revisit each house 4 hours after the second measurement and the day would be too long otherwise). The communities had to be reasonably close to one another to allow cars to drop off the census taker as part of the advance team in one community, and the survey team in another. Each community had a census of all households with children aged between one and seven years. Fifty households per community were randomly selected to participate. One child within the age range was randomly selected within the household. No household or child refused the study. Older children were not eligible for the study because they had to have their faces washed for school in the morning and would not be available for the observation later in the day. One community was randomly selected as a control, to monitor facial status over the course of the day in the absence of face washing. All children in the study were enrolled for one day in September/October 2022.

In November 2022, the children who had previously served as controls were revisited for one day. For this visit, we adhered to the intervention protocol and the mothers washed the children’s faces immediately after the baseline set of observations. The purpose of this visit was to ensure the control children were no different than the intervention children at least in response to face washing.

### Study implementation

A 3-day study training program was instituted at the start to provide an overview of the study and to train team members on observing clean faces and observing the facial wipes. The reliability study was carried out as described above. After the reliability study, the team members were each assigned a role, with the face grader with the highest kappa compared to MK and the wipe grader with the highest score compared to MW assigned to those roles. In each of the four communities a census taker went in advance to explain the study to the community leaders and get permission to approach the community members. A complete census was undertaken, and households with children age under seven years were eligible for participation. One eligible child was randomly selected from each household to participate. The census taker explained the study at each household, and in particular explained that if the mother wished to participate, to not wash the child’s face before the team arrived on the scheduled date. The guardian was told the child would be washed that day as advised by the team.

The survey team had a schedule of ten households to visit in the morning, and four hours later in the afternoon. This schedule accommodated observing the child and facial wipes pre-wash, having the child washed by the guardian, then immediately observing the child and the facial wipes post wash. Four hours later, with no intervening washing, the child and facial wipes were observed again. In the households in the control community, the guardian was asked to refrain from washing the child until after the team had completed both the morning and afternoon observations.

The team approached the household, explained the study, and gained informed, written consent from the guardian of the child. A facial cleanliness observation was made, then a facial wipe was taken of the right eye according to protocol as stated above. The wipe was graded immediately as per protocol. In the control village, the baseline observation was immediately followed by the second facial cleanliness observation and a wipe of the left eye. In the other villages, after the baseline observation, the mother was asked to wash the child’s face as she usually does, using either a clean rag, water, or water and soap. The team provided water for washing. After washing, the child was again observed for facial cleanliness, and a wipe taken of the left eye. In all communities, the mother was instructed not to wash the child until after the team returned in the afternoon, about four hours later. The last set of observations consisted of observing facial cleanliness, taking a wipe again of the right eye, and observing the wipe and assigning a grade.

### Sample size

We assumed that the power calculation would be driven by the percentage of clean faces. With 50 children in the control community and 150 in the other communities, assuming that 50% of the children in the control community had clean faces, using a two-tailed α level of 0.05, we have 81% power to detect a 1.44 relative risk (p1 = 0.72)

### Data analyses

Reliability was assessed using the kappa statistics, with a weighted kappa for the comparison of color grade assigned to the facewipe. For the cross-sectional component, the Fisher’s exact test was used to examine the strength of the association between the proportion of clean faces in the control community versus the proportion in the other communities. Differences in the distribution of the color grades between the two groups was assessed using an unpaired two-sample Wilcoxon test because the distribution of grades was so narrow.

For the comparison between observations over a day of no face washing and a day with a face washing in the children in the control community, the McNemar’s test for correlated proportions was used to examine the strength of the association between clean faces. The Wilcoxon Signed Rank Test was used to test for differences in the distribution of color grades.

All analyses were carried out using SAS version 9.4 (Cary, N Carolina)

## Results

The preliminary study found the grade for the wipe increased with the darkness of the dirt, range from grade 3 (darkest) to grade 8 (whitest) independent of the amount of dirt actually on the hand.

There were 91 children and seven graders who participated in the study of reliability of grading faces for cleanliness and face wipes for degree of darkness. Five graders observed 91 children twice. One grader observed and recorded 86 children the first time (she forgot to record 4 grades and one child was crying so not eligible)and 91 the second time (86 paired observations). One grader observed 90 children the first time and 90 children the second time (89 paired observations). The intragrader reliability for each grader was based on the number of children observed twice by each grader; the intergrader reliability for each pair of graders was based on the maximum number of children that had grades from both graders (21 kappa agreements total). The intragrader reliability for observing a clean face was kappa = 0.64, range of 0.47–0.78 (Table 1). The intergrader reliability was 0.53, kappa ranged from 0.36–0.75.

**Table 1:** Intra and inter grader reliability of seven graders in observing the presence of a clean face, and grading a facial wipe in 91 children in Kongwa Tanzania.

	Percentage with clean face/average score on wipe according to observer with highest intragrader kappa	Average Kappa value	Range of scores
**Observing a clean face**	79%	-	-
Intra grader agreement	-	0.66	0.57–0.87
Inter-grader agreement	-	0.53	0.36–0.75
**Observing color of wipe**	8.0 (standard deviation = 0.7)		
Intragrader agreement	-	0.57	0.40–0.75
Intergrader agreement*	-	0.52	0.36–0.76

* One grader was found to have copied answers from another grader and her results are not included.

All seven graders graded the color wipe twice, which was obtained on all 91 children. The intragrader reliability was weighted kappa = 0.57, range of 0.40–0.75. The intergrader reliability, excluding the one grader who was copying answers, was 0.52, with a score ranging from weighted kappa of 0.36–0.76. Overall, an estimated 79% of the 91 children had clean faces, with an average color wipe score of 8 (Table 1).

The comparison study of the two measures of a clean face, face wipe and observation of a clean face, was carried out in 202 children whose average age was 3 years (Table 2). Females were 55% of the control children and 59% of the children who were cleaned after the first observation.

**Table 2:** Characteristics of the sample of children in the comparison of two measures of a clean face.

Characteristic	children in control village N = 51	Children in villages randomized to face washing after first observation N = 151
Age in years		
Mean age in years (SD)	3.4 (1.6)	3.2 (1.6)
1 year, n(%)	7 (13.7)	29 (19.2)
2 years, n(%)	10 (19.6)	24 (15.9)
3 years, n(%)	13 (25.5)	31 (20.5)
4 years, n(%)	6 (11.8)	27 (17.9)
5 years, n(%)	9 (17.7)	29 (19.2)
6 years, n(%)	6 (11.8)	11 (7.3)
Sex		
Female, n(%)	28 (54.9)	89 (58.9)

The pre cleaning values of all children were used to evaluate the correlation between the presence or absence of a clean face and the grade of the facial wipe. There was no association. Those with a clean face had a mean face wipe grade of 6.7 (SD = 0.68), while those with unclean face had a mean face wipe grade of 6.6 (SD = 0.78) (p = 0.75).

The children who were not washed over the study period did not have a significant difference in the proportion of clean faces observed at each time point (Table 3); at baseline 47% had clean faces, and 4 hours later 48% had clean faces. Among the children whose mothers washed their children after the pre-cleaning time point there was a significant improvement in observed clean faces immediately after washing, from 50%-88%. The clean faces persisted 4 hours later, with 65% having a clean face, compared to 48% in controls, a significant difference (p = .03)

**Table 3:** Proportion of observed clean faces in children enrolled in the control and washed child study group at each time point.

	Control N = 51 children	Children cleaned after baseline observation N = 151	
Observation time	n (%)	n (%)	Cleaned vs control p value
BASELINE:Pre-cleaning n (% clean)	24 (47.1)	76 (50.3)	0.69
1^st^ FOLLOW-UP Immediately after baseline n (% clean)	27 (52.9)	133 (88.1)	.0001
2nd FOLLOW-UP 4 hours after 1st follow-up, n (% clean)	24 (48.0) *	98 (64.9) *	.03

*One child was not available in each group at the 4 hour follow up.

Similar analyses were conducted comparing the data from the grading of the face wipe in children in the control community to the children in the others whose faces were cleaned after the first observation (Table 4). There was no difference in the two groups at baseline, but a significant difference in the means immediately after facial cleaning, with less dirt on the wipes from the cleaned children, mean 8.0, compared to the control children, mean 6.7. Although the cleanliness declined after four hours, a significant difference persisted, with a control mean of 6.7 versus a mean of 7.1 in the children who had been washed four hours earlier.

**Table 4:** Mean and median of face wipe grade* in children in the control and washed child villages at each time point.

Observation Time	Statistic	Control N = 51 Children	Children washed after baseline observation N = 151
BASELINE:Pre-cleaning	Median (IQR)	7 (6–7)	7(6–7)
	Mean (SD)	6.6 (0.7)	6.7 (0.7)
	Range	(5–8)	5–9
	p-value**	0.70	
1^st^ FOLLOW-UP:Immediately after baseline	Median (IQR)	7 (6–7)	8 (8–8)
	Mean (SD)	6.7 (0.8)	8 (0.7)
	Range	(5–8)	6–9
	p-value*	< .0001	
2^nd^ FOLLOW-UP: 4hours after 1st follow-up	Median (IQR)	7 (6–7)	7(7–8)
	Mean (SD)	6.7 (0.7)	7.1 (0.7)
	Range	(5–8)	5–9
	p-value*	0.001	

*Grade ranges from 0 (black) to 10 (pure white)

**Wilcoxon two sample test

We sought further reassurance that the control children were similar to the children who were cleaned after the baseline observation. We observed the control children using the same 3 observation points again one month later, but this time having their mothers wash their faces after the baseline observation. (Table 5). Two of the children were not available one month later, so the paired comparison was conducted with 48 children. There was no difference in the distribution of children who were observed to have clean or unclean faces prior to washing at baseline compared to one month later, and in fact it was not the case that all the children observed to be clean were those clean again at the one month baseline visit (paired test p = 0.4) As expected, immediately after face washing the children all had clean faces observed compared to the observation of clean face when no washing was done. At the 2^nd^ follow-up visit the proportion with clean faces was 50% when they had not been washed previously and 65% when they had been washed previously; evidence of a decline after four hours but not to the level seen without washing. The difference was not statistically significant.

**Table 5:** Paired comparison of 48 children who had no face washing at baseline (control) but had faces cleaned after initial observation one month later. Clean Face observations.

Observation time		Observations one month later with face washing between Baseline and first follow-up		
	Control observations (no face washing between first and second time point)	Clean	Unclean	P value
BASELINE	Clean	14	11	0.41
	Unclean	13	10	
1^st^ FOLLOW-UP	Clean	22	0	<0.0001
	Unclean	26	0	
2^nd^ FOLLOW-UP: 4 hours later	Clean	14	10	0.13
	Unclean	17	7	

The same comparison was made for the face wipe grade. We found that the two baseline observations (prior to washing) suggested the same children had a cleaner face wipe score one month later compared to their face wipe score previously. The mean difference in score was 0.29, p = 0.02 (Table 6). This significant difference could indicate a secular trend in cleanliness of children or a trend in higher scores in grading over time, although the period of time between assessments was just one month. As expected, there was a significant increase in the score graded for the face wipe after face washing compared to the score graded in the same children in the absence of face washing, an increase of 1.94 or almost 2 grades. The change in the face wipe grade also declined after four hours from face washing but was still statistically significantly higher than the grade in the same children without washing.

**Table 6:** Paired comparison of 48 children who had no face washing at baseline (control) but had faces cleaned after initial observation one month later. Observations on Face Wipes.

	Difference in face wipe grade (follow up -baseline)			
Observation time	Mean (SD)*	Median (IQR)**	Minimum to Maximum value	P value
BASELINE	0.29 (0.85)	0 (0–1)	-1 to 2	0.02
1^st^ FOLLOW-UP	1.94 (0.95)	2 (1–3)	-1 to 4	< .0001
2^nd^ FOLLOW-UP: 4 hours later	0.77 (0.97)	1 (0–1)	-1 to 3	< .0001

SD = standard deviation

IQR = interquartile range

## Discussion

The purpose of this study was to compare the performance of two measures of a clean face. One measures the observation of ocular and/or nasal discharge on the face, and the other measures the degree of dirt on the face. There was no correlation between the two measures. In part, this may be due to the variability in the color of dirt, as darker dirt would give a higher score than the same quantity of lighter colored dirt on the same face. In part it is also because the measures are not assessing the same metric. Ocular and nasal discharge may be present on an otherwise clean face if the eyes and nose were not specifically wiped, and wiping discharge with a dirty cloth may leave residual dirt on the face.

For both measures, the reliability among and between graders was similar. The agreement between graders in observing a clean face was lower than has been reported (2), and maybe due in part to mothers cleaning the child’s face midway in the exercise despite being asked not to do so. The exercise was carried out in a central location, and being around other mothers may have resulted in feeling peer pressure to clean the child. We found a higher proportion of children with clean faces (and a higher face wipe grade) during the reliability test than when we did the observations at the home, providing further support for the possible peer pressure effect of doing the reliability study at a central location. This finding was similar to an earlier finding comparing clean faces at a central site versus at the home [5] The relatively low kappa agreement, even weighted, in grading the face wipe was also lower than reported elsewhere (4). Comments from the trainer and trainees suggest the difficulty seemed to arise from determining the area around the darkest patch on the wipe, especially if there were multiple dark patches. This could arise if there was shifting pressure between fingers during the wiping process.

For the cross-sectional comparison, the group that later had faces cleaned was no different in either the proportion of clean faces or the grade on the face wipe compared to the control group at the first measurement time. This similarity provides reassurance of the comparability of the two groups, and that changes over time are likely to be real. Both measures of clean faces were sensitive to the face cleaning of children compared to control children, with significant differences in the proportion of clean faces and improvement in the face wipe score measured after cleaning. After four hours, some loss in cleanliness over time was expected in the group of children whose faces were washed, but they still were significantly different from the control children with a higher percentage of clean faces and higher face wipe score. We were concerned that the face wipe score might be artificially high at 4-hour observation because the same eye was wiped pre-cleaning and again at 4 hours. Rather than a reflection of the face washing, the cleanliness could be a reflection of the use of the facial wipe itself. However, the fact that in the group of control children who were not cleaned, the baseline and 4-hour observation of the facial wipe were similar (see Table 3) suggested the impact of using the wipe itself was minimal.

The findings on some loss of cleanliness but not to pre-washing levels were interesting because the children in the study were the youngest ones, and immediately after the team left the house, were observed to go back to playing in the dirt around the household. The mothers were specifically asked not to wash the child again until after we returned 4 hours later, but it is possible some mothers performed an interim face cleaning on the child. We felt this was a relatively rare event because there was no evidence the mothers of children in the control community cleaned the children during the four hours and they would have had as much incentive to do so as the mothers of children who were cleaned.

We repeated the observations on the children in the control community one month later, only this time asking the mothers to wash the children after the first observation. This paired comparison would enable us to see if the children in the control community did behave similarly to the children in the communities where faces were cleaned after the first observation. We found that the proportion of children with clean faces at the first observation, 52%, was statistically the same, 56%, as at one month later. However, the grade of the face wipe was significantly higher at one month in the same children compared to their value previously, a difference in the score of the two of 0.29. Because the facial cleanliness grader was different from the face wipe grader, and the two observations were masked from each other, we do not feel the improvement over time was a secular trend in grading. Rather, the improvement may be due to circumstances in the households or community. Since only one month separated the observations, it is unclear what those circumstances may have been. The team reported no noticeable changes in the environment had occurred in the community. It is possible that some mothers washed their child’s face prior to the baseline visit in the second round, despite being asked not to.

The finding of differences in the baseline measurements does mean that the measurements finding improved cleanliness comparing the first set of measurements to the second set one month later must take into account that there were unexpected differences at baseline in levels of cleanliness as well. Nevertheless, there was a significant improvement in the proportion of clean faces and in the face wipe grade immediately after cleaning, compared to the respective metrics in the same children with no cleaning. This is not surprising and expected. The magnitude of change was higher than expected, with 100% of children observed to have clean faces and a score on the face wipe that increased by almost two grades. In part some of this may reflect the secular trend in cleanliness observed at baseline, but not all. The observations made 4 hours later also showed that some effect of cleaning the child’s face persisted, with 56% of children still clean after washing compared to 50% in the same children in the absence of washing. The absence of statistical significance may reflect the sample size of 48 children; we would have needed 181 children to detect the difference observed. The face wipe grade was statistically significantly higher at 4 hours post cleaning compared to the face wipe grade in the same children at 4 hours without cleaning, with a difference of almost one grade. Because the results from our paired comparison of the control children were similar to the results in the study comparing the control children to the 151 children who had their faces washed after baseline, we feel that the children in the control community were not very different from those in the communities where face cleaning was carried out initially.

There were limitations to the study. Due to logistic considerations, we were not able to measure facial cleanliness longer than four hours after cleaning. It is possible that there would be differences in the two metrics if we had been able to study the aspect longer. Also, we observed some mothers cleaning the child’s face between round one and two of the reliability study, so the agreement within and between graders was not as high as it would have been if no cleaning had been done. We did not have field experience training the graders on grading the face wipes and felt we did not allocate adequate time for this, despite adding an afternoon and evening training to the original day allotted. We allowed the mothers to clean the child’s face as they usually would, and in some cases, it was clear the eyes or nose were not targeted, or that the child would not permit time to get all the dirt off. This may have affected the observation immediately after cleaning, and why the scale on the face grade was not fully used. We noted that although the scale for grading the face wipe went from 0–10, and was set up to be quantitative, in fact rarely were any scores outside of 6–8. We recognize that the for the control group, the face and wipe graders may have remembered the previous grade they assigned at baseline and thus the agreement between baseline and immediate first follow up was tighter than it might have been if two graders had carried out the grading.

In summary, both metrics for measuring a clean face had similar measures of reliability, and both detected change in facial cleanliness that resulted from cleaning a child’s face. Both showed an ability to detect the change immediately after cleaning and four hours later, even with a decline in facial cleanliness over the four hours. In our experience there were both positive and negative considerations with each method. In terms of training, it was hard for trainees to learn how to pick a spot on the wipe to grade, especially with multiple dark spots, and training took longer than for observing the presence or absence of a clean face. In terms of cost, the simple observation of a clean face and the observation of a facial wipe both require personnel training; however, there is the additional cost of the procurement of sterile saline wipes, transport to the field, and proper disposal of the wipe and the packet. The use of a binary variable, face clean or unclean, may not be sensitive to incremental change compared to a scale of eleven points. In reality though, the extreme values were never used and the scale ranged largely from 6–8 with an occasional 5 or 9. Nevertheless, differences of less than a grade were detected which suggests that a smaller sample size may be possible to detect change when using the facial wipe. However, such a small change has unclear meaning as our data suggest that face washing results in at least 1–2 unit change in face wipe grading to reflect the behavior, and a 17–30% difference in the percentage of clean faces. These are big changes. Our observations from the paired analyses suggest differences of less than a unit with the facial wipe, or less than 10% in observation of clean faces may reflect noise in grading rather than true, population based, behavior change.

As noted before, the metrics measure different aspects of a clean face. If dirt is the metric of interest, then observing a clean face as defined here will not reliably capture that aspect. Similarly, if ocular and nasal discharge is the metric of interest, then a facial wipe will not reliably capture the absence of those elements. Both measurements appear to capture the behavior of facial cleaning, and the choice would appear to rest on the measurement that captures the stated objective of the behavior, consideration of costs, training, logistics, and implementation.
